# Measuring mental health in humanitarian crises: a practitioner’s guide to validity

**DOI:** 10.1186/s13031-021-00408-y

**Published:** 2021-09-26

**Authors:** Brandon A. Kohrt, Bonnie N. Kaiser

**Affiliations:** 1 George Washington University, Washington, DC USA; 2 University of California San Diego, La Jolla, CA USA

**Keywords:** Validation, Complex humanitarian emergencies, Mental health and psychosocial support, Assessment, Psychometric properties

## Abstract

**Background:**

There are ongoing methodological advances in measuring mental health in humanitarian crises. This Special Section describes numerous innovations. Here we take a practitioner's view in understanding the key issues related to assessment of mental health in humanitarian contexts and how the innovations contribute to the field.

**Main body:**

In this guide for practitioners, we address the following issues: (1) clarifying the intended purpose of conducting mental health assessment in humanitarian crises: why is this information collected and for what intended purposes?; (2) determining what type of tool should be selected and the types of psychometric properties that are important for tools serving this particular purpose; (3) when a validated tool is not available, considering how qualitative and quantitative methods should be used to generate information on validity; and finally, (4) how to report on validity and its implications for interpreting information for humanitarian practitioners, governments, care providers, and other stakeholders supporting people affected by humanitarian emergencies.

**Conclusion:**

Ultimately, mental health assessment tools are not independent of the group with which they were designed, nor are the psychometric properties of the tools or their utility universal across purposes. Therefore, organizations and stakeholders will optimize their positive impact when choosing tools wisely, appropriately adapting and validating tools, and providing guidance on how to interpret those findings to best serve populations in need.

## Introduction

One of the challenges for humanitarian organizations working in the field of mental health and psychosocial support (MHPSS) services is ensuring that the people who most need care and support are receiving it and that the services delivered are improving mental health and psychosocial wellbeing in a meaningful way. This Special Section on valid psychological assessments in *Conflict and Health* has explored a number of rigorous and cutting-edge approaches to questions of validity of MHPSS tools in humanitarian settings [24].

In this commentary, we provide a guide for humanitarian organizations to optimize validity for a particular context and measurement objective. We build upon the contributions of specific articles as well as summarize current best practices in the field. This commentary is presented in 4 sections: (1) clarifying the intended purpose of conducting mental health assessment in humanitarian crises, i.e., why is this information collected and for what intended purposes; (2) determining what type of tool should be selected and the types of psychometric properties that are important for tools serving this particular purpose; (3) when a validated tool in the setting for the intended purpose is not available, how to conceptualize and implement qualitative and quantitative methods to generate information on validity; and finally, (4) how to report on validity and its implications for humanitarian practitioners, governments, care providers, and other stakeholders supporting people affected by humanitarian emergencies. We predominantly focus on assessment tool validity in relation to psychosocial distress and mental illnesses in humanitarian settings, but we also draw attention to the gaps and challenges related to tools that capture positive states of mental health and resilience.

Determining validity of assessment tools and correctly interpreting that information is important because of potential negative consequences when tools are inappropriately used or results inaccurately interpreted [15, 17, 30]. At the individual level, a tool has the potential to miss those who need care or the potential to inappropriately label someone who does not have a disorder, which leads to stigmatization and burden on service providers. This highlights one of the grey areas in humanitarian mental health and other low-resource settings where the lines are blurred between screening tools and diagnostic tools. Screening tools are intended to determine the *likelihood* of someone having a disorder, which is then confirmed by a mental health professional’s evaluation. Some mental health professionals use a diagnostic tool to make or confirm a diagnosis. Screening tools are typically self-report or completed by an interviewer who is not a trained clinician, whereas diagnostic tools are completed by mental health specialists, who have also received training on the particular diagnostic tool.

However, in humanitarian mental health and some programs in global mental health, screening tools have become de facto diagnostic tools because of too few mental healthcare providers to follow up a positive screening with a diagnostic evaluation in emergencies and other low-resource settings [25]. Although ideally, screening tools should not function as diagnostic tools, the fact that they continue to be used in this manner makes it vital to ensure strong validity. At a population level, tools with inappropriate validity risk underestimating the population burden, which leads to a lack of resources mobilized. Conversely, a tool may overestimate the burden, leading to deployment of services and resources to persons who do not need care, which detracts from resources needed in other humanitarian sectors. Often, tools that have not been adequately adapted and validated may lead to patterns of bias where–at the individual and population level–particular ethnic, linguistic, socioeconomic, gender, and other groups are over-represented and others are underrepresented among those identified with a mental disorder [8]. This can be a major contributor to ethnic, racial, and gendered disparities in care. Therefore, it is vital to clearly articulate the purpose for using a tool, establish adequate validity for the intended purpose, and transparently report on how validity influences the results of a tool’s use in a particular humanitarian setting.

## Part 1: What is the purpose of mental health assessment?

The first issue to clarify is the intended purpose of the mental health assessment [15]. The purpose will influence what type of instrument is selected, what psychometric properties to consider, and what adaptation and validation need to be done. Because humanitarian emergencies typically require instruments to be readily available or quickly adapted, we will focus on more rapid approaches, although more extensive approaches are optimal when resources of time, funding, and expertise allow.

### Building your team

The first step is to establish an appropriate team representing key stakeholders for the intended use. This should include representatives of affected populations (i.e., service users including persons with lived experience of mental illness), members of implementing humanitarian organizations, local mental health professionals, and experts in qualitative and quantitative methods of adaptation and validation. This team composition will help ensure that the questions will be relevant to beneficiaries and that academics and other consultants are in a position to encourage addressing problems in a way that can have real-world application and benefit. The conversations within this team can raise questions about what types of data would need to be collected to answer important questions. Useful examples of this are the Special Section paper on a depression screening tool in Haiti in which validation led to cut-off scores that can be directly applied to screening school children for MHPSS programs [18]. Similarly, the network analysis on Sudanese MHPSS symptoms and programmatic needs can directly inform what services would have greater benefits [21].

### Concepts and terminology

Before discussing the purpose of a mental health assessment, it is important to have clarity around terminology for validity. Understanding these concepts is relevant when determining if a tool fits the intended purpose and to judge potential tradeoffs of what a tool does well versus its shortcomings. **Validity** is the concept that a tool captures a real-world category in some way [30]. Therefore, validity is a relative quality, i.e., valid in relation to some other form of categorization or identification. A valid mental health assessment tool means that the outcomes of an assessment tool have some resemblance to what a trained mental health clinician would determine if they evaluated the individual; this is typically criterion validity against a clinician’s diagnosis [30]. This type of validity is typically established by having individuals complete an assessment tool and then individuals being evaluated by a mental health clinician, who provides a diagnosis using a structured clinical interview. Commonly assessed disorders in humanitarian settings are major depressive disorder, posttraumatic stress disorder (PTSD), general anxiety disorder (GAD), and substance use disorder, and there are increasing calls to assess severe mental illnesses such as bipolar affective disorder and psychosis disorders [2]. Therefore, a valid tool, in this sense, is one where results of the assessment tool are similar to the clinician’s structured diagnosis.

A range of information is needed to make determinations related to validity [15]. A **true positive (TP)** is an individual who scores above a specific cut-off on an assessment tool, and that same individual receives a diagnosis for the condition of interest by a clinician. A **true negative (TN)** is an individual who scores below the specific cut-off on an assessment tool, and that same person is determined not to have the condition by the diagnosing clinician. Ideally, a valid tool would have only true positives and true negatives, which suggests a perfect matching of the tool’s information and clinicians’ assessments. However, that is rarely the case in mental health. Instead, there are often **false positives (FP)** on assessment tools (Gilbody et al. 2007; Thombs 2012). These are individuals who endorse symptoms above the cut-off point on a self-report scale, but when evaluated by a clinician, they do not meet diagnostic criteria. Conversely, most cut-offs will also produce a certain number of **false negatives (FN)**. These are individuals who score below the cut-off, but the clinician determines that they do have the diagnosis of interest. False positives and false negatives can occur because of differential interpretation of the wording in a tool, not endorsing items because of stigma related to mental health, endorsing items because of expectation of services and benefits, and subjective differences in experience and interpretation.

When validating a tool with the clinician’s diagnostic interviews, the true positive, true negative, false positive, and false negative can be used to calculate psychometric properties to describe the tool’s validity [22]. **Sensitivity** can be understood as the proportion of respondents who are true positive (i.e., truly experience the condition of interest) out of all the diagnosed positive participants, i.e., Sensitivity = TP/(TP + FN). The fewer the false negatives, the closer to 100% sensitivity. An ideal tool to identify people in need of care would have 100% sensitivity. However, sensitivity needs to be considered in light of a tool’s specificity. **Specificity** refers to the proportion of respondents who are true negatives (i.e., truly *do not* experience the condition of interest) out of all participants who are not diagnosed by the clinician, Specificity = TN/(TN + FP). The fewer the number of false positives, the closer to 100% specificity. For any tool, the cut-off score will have a certain sensitivity and specificity. Selecting lower cut-off scores can increase sensitivity, i.e. more people with a condition will be captured because there are fewer false negatives. But aiming for a higher sensitivity by using lower cut-off scores often leads to the trade-off of poor specificity; this means that there will be more false positives (i.e., more people falsely identified or diagnosed).

A related concept is **positive predictive value (PPV)** [22]**.** This is the likelihood that someone with a score above the cut-off is likely to have the condition. The positive predictive value is the proportion of true positives out of all persons scoring above the cut-off, which also includes false positives; PPV = TP/(TP + FP). Conversely, **negative predictive value (NPV)** is the likelihood that someone with a score below the cut-off does not have the condition of interest. This is the proportion of persons who are true negatives compared to everyone with a negative test, which also includes false negatives, NPV = TN/(TN + FN). Overall **accuracy** of a cut-off on a mental health assessment is the total number of people correctly classified compared to the total population of interest.

The sensitivity, specificity, PPV, and NPV are based on the characteristics of the sample on which the validation study was done. The sensitivity and specificity are influenced by the age of the population, burden of stressors and trauma, and local cultural differences [8]. Similarly, the sensitivity and specificity can vary based on whether this is a treatment-seeking clinical population vs. community population because of the types and severity of symptoms that may be present [18]. In addition, the PPV and NPV need to be adjusted based on the prevalence in the population [1].

### Tool properties for clinical services and other interventions

There are two general reasons that mental health assessments are used in humanitarian emergencies. The first is assessing needs for engagement in services. These services can be clinical treatment, psychosocial support programs, mental health promotion or prevention, livelihood support, education, and other social services. The type of service, availability, costs, and consequences if services are not received all influence how sensitivity, specificity, and other psychometric properties should be considered [15]. As mentioned above, the ideal tool would be 100% sensitive to ensure that everyone who needs the services gets them, and 100% specific to be sure that persons who do *not* need services are *not* enrolled in the programs. This ‘perfect fit’ model means that there will not be anyone who goes untreated. At the same time, it ensures that resources (time, funding, human expertise) are not diverted to persons who do not need the services. Moreover, when conditions may carry a stigma—as is often the case with mental illness—it also means that people are not inappropriately labelled, leading to unintended negative social, economic, or educational consequences [14].

**Fig. 1:**
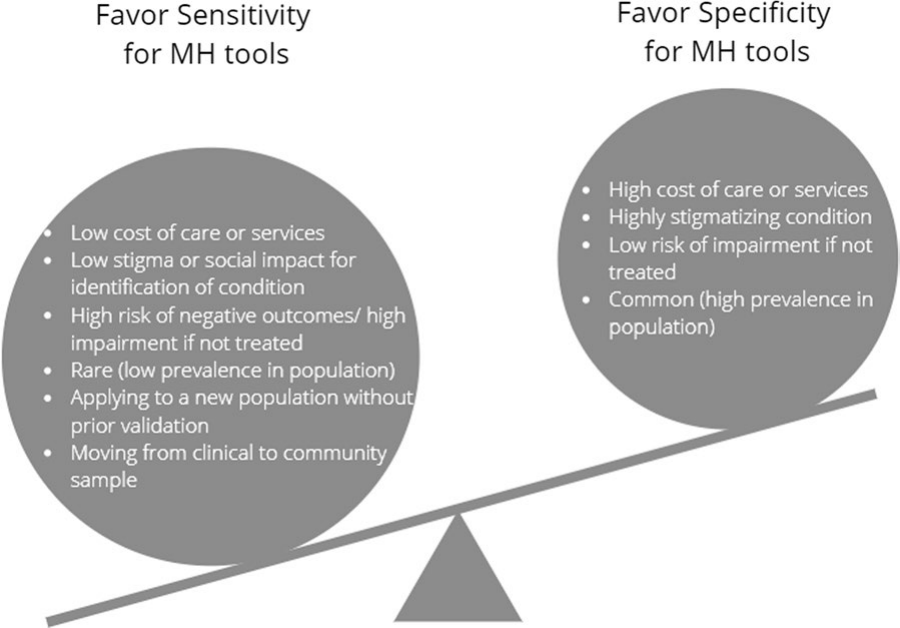
Considerations for prioritizing sensitivity vs specificity of assessment tools

But tools rarely have this perfect fit. Therefore, there are a number of considerations to decide what balance of sensitivity and specificity should be sought (Fig. 1). More sensitivity is important when there are serious longer-term consequences if care is not provided. Sensitivity should be high when there are associated adverse risks to one’s physical health and safety, as well as that of family, partners, and children. Specificity becomes important to consider in regard to resource burden and potential negative consequences. For example, in a humanitarian setting where there may be few mental health professionals or other forms of treatment, a poor specificity means that there will be a lot of ‘false positives’ burdening the already taxed health infrastructure. Another consideration for specificity is the social and economic consequences of potentially labeling someone. If the condition of interest is anxiety or depression, the risks of ‘false positive’ status (i.e., labeling someone as depressed when they are not clinically depressed) is not likely to be as severe as if the screening were for a psychosis diagnosis.

The number of steps in screening and enrollment also is a consideration. If there is an additional step in evaluation before enrollment in treatment or an intervention program, then a high sensitivity with low specificity would be permissible because the secondary evaluation can exclude false positives. However, if secondary evaluations are costly in-and-of-themselves, or if there is not an additional evaluation after the assessment tool, then at least modest specificity is needed.

With regard to treatments and programs, if there is a relatively low cost per individual, then high rates of false positives do not create a large financial burden. However, if the intervention costs per person are high, then large numbers of false positives can financially overwhelm the program. Interventions that are delivered on a group-level, school-level, or community-level typically have lower costs and can accommodate false positives. The type of intervention makes a difference with regard to potential benefits and harms. If the intervention is a mindfulness-based stress reduction or problem-solving skills, this is unlikely to have negative effects, and it can benefit participants who may not have a clinical-level disorder. For example, in the school-based validation of the Zanmi Lasante Depression Symptom Inventory, the researchers identified a cut-off with 100% sensitivity and 74% specificity [18]. Even though this would lead to inclusion of false positives, the false positives are unlikely to experience harm and may even benefit from low-cost school-based interventions. If, however, the treatment is medication-based with potential side effects, then false-positives have the risk of side effects without any of the benefits because the person does not have the condition. Studies of anti-depressant medication, for example, have shown that persons with mild depression experience limited benefit, whereas ensuring that persons with moderate to severe depression have access to appropriate medication can dramatically improve quality of life [4].

### Tool properties for prevalence and population-based studies

In addition to identifying individuals in need of care, the second purpose of mental health assessment tools in humanitarian settings is for population-based studies. This is typically to assess the burden of mental health problems in a population after a humanitarian emergency [2]. This information is used to determine allocation of resources for populations most in need. This can also be used to monitor changes over time in prevalence to detect upward or downward trends, as well as how these vary by setting and group. Population-based studies have been used to detect increases in mental health problems in response to the COVID-19 pandemic, for example a recent study in Bangladesh [20]. There are different ways to consider psychometric properties for populations. If a categorical percent is needed (% of the population with depression), then sensitivity and specificity again need to be balanced. Cut-offs with high-sensitivity and low specificity will substantially overestimate the burden and have high rates of false positives. This can present an inaccurate picture and lead to resource allocation that is disproportionate to the need. Typically, because the percentage of persons with mental illness–even in conflict affected settings–is around 10–20% [2], a low specificity can mean that for every 1 person with a condition, 2 false positives are included. Therefore, for population-based studies, it is important to have a reasonable specificity.

## Part 2: Selecting the right tool

Now that you have **clarified the purpose of the assessment**, the next set of questions relate to how to select, prepare, and validate a tool in the most efficient and informative way possible, recognizing the constraints of working in humanitarian contexts.

### Select the tool

A key task is to select the assessment tool(s) that you will use. Your overall purpose will help guide this process, particularly if there is a disorder of focus (e.g., depression or PTSD) versus examining general experiences of distress or wellbeing. You should begin by searching for existing tools, especially those that have previously been rigorously adapted, validated, and used in similar populations. The Global Mental Health Assessment Database (GMhAD) could be a starting point, as it identifies mental health assessment tools that have been adapted and/or validated for use in low- and middle-income country contexts (https://global-database.thefpr.org/https://global-database.thefpr.org/). Identifying an existing tool does not preclude the need for additional work. For example, Legha and colleagues [18] used the Zanmi Lasante Depression Symptom Inventory in their study of school-aged youth in Haiti. Although the tool had already been locally developed and validated, that was in a clinical population, and they found that a different cut-off score was appropriate for their community-based sample. If you do not identify tools that have been used in similar populations, you could consider adapting an existing assessment tool [31] or developing a novel tool (e.g., [7, 9, 23]). Another important consideration is selecting tools that may reflect positive mental health or contribute to resilience, such as Getnet and Alem’s [5] study on coherence in this Special Section.

## Part 3: Adaptation and validation

We will describe procedures for validating tools, but it is important to note that validation cannot be performed rigorously—to provide confidence in the findings and their local applicability—without the complementary process of qualitative adaptation. Although we describe these steps as though they occur in a certain order, the process is rarely linear, as these decisions are typically made and revisited in a more iterative manner.

### Determine validation strategy

You should specify the strategy for conducting a validation study, which will inform the specific psychometrics and type of validity that are relevant. The most common approach is clinical or diagnostic validation, which compares an assessment tool against diagnoses provided by clinical professionals in order to assess its performance and select an appropriate cut-off score. Clinical gold standards are typically what one thinks of when stating ‘validity’, but alternative strategies to clinician diagnoses are also available. Perhaps clinical diagnoses are less relevant than comparing a tool’s performance to local categories of distress or functioning. In another context, test–retest reliability^1^ might be the most important psychometric, such as for tools that are administered repeatedly to gauge effectiveness of an intervention.

### Select sample

Although you likely already have an affected population in mind, you will need to select a sample for your study, or the group/s that will represent the affected population. The study sample should not be meaningfully different from the general affected population. For example, if you aim to use your assessment tool for community-based detection, it would *not* be the ideal strategy to select a clinical population to validate the tool. Similarly, the sample should be similar to the affected population in terms of socioeconomic status, age, language/s spoken, etc. That is, the sample should be representative of the target population. If there are multiple groups—which is often the case within a humanitarian conflict or across multiple humanitarian settings—then you will need to consider other confounds for mental health needs (e.g., forced migration, trauma exposure, poverty, minority status). It is important to recognize and account for meaningful heterogeneity between groups [10, 28]. Particularly where there are multiple affected groups, it is important to consider who will be conducting data collection so that there are similar relationships between enumerators and affected persons across groups.

### Qualitative work

Below, we describe a mixed-methods approach to culturally adapt assessment tools for use in new settings. However, you should also consider whether there are additional areas where qualitative research could inform assessment tool preparation. For example, you might use rapid assessment within the target population to identify needs and select tools. Getnet and Alem's [5] focus on positive psychological attributes was likely informed by prior qualitative research. You might also identify relevant idioms of distress or other cultural conceptualizations that are key to communicating about and identifying those experiencing mental distress (e.g. [9, 11, 14, 26]). These qualitative findings would likely lead to refinement of the list of tools selected according to needs in the target population. It is common practice to hire qualitative research experts as consultants to conduct these sub-studies. We recommend a set of questions to be sure to discuss with consultants in such scenarios (Table 1).

**Table 1:** Topics to discuss with qualitative consultant

What is the main qualitative research question?	This should be a “how” or “why” question rather than focused on measuring or counting
How will the qualitative findings be used? What is the goal?	Identifying items for a survey is much faster than developing a detailed description of mental health concepts or healthcare decision-making
Are there sub-groups whom you anticipate will have different experiences from each other (e.g., linguistic groups)?	This is important to know in planning the sample size and recruitment strategy
What forms of data collection might be possible?	If there are existing mental health services, observation or review of clinical records could be used, in addition to interviews and focus group discussions

### Conduct transcultural translation and adaptation

As mentioned above, if you cannot identify tools previously validated for the affected population, you will most likely select and adapt existing screening tools. van Ommeren and colleagues [31] describe a process for transcultural translation and adaptation of tools that has been widely applied. The approach begins with multiple translations by lay and professional individuals. Central to the approach is establishing equivalence of initial and translated items through exploring comprehensibility, acceptability, relevance, and response options through a series of focus group discussions with members of the target population. Data from focus groups inform further adaptation to ensure equivalence and completeness of items. Compared to typical approaches that use translation/back-translation, this process involves more in-depth data collection within communities. Significantly, local participation expands beyond researchers, mental health providers, or others who may have undergone higher education or take a slightly different approach to identifying and understanding problems than affected persons. This broadening of perspectives is critical to ensure that expertise of affected populations is incorporated and that appropriate translations are used—both in terms of accuracy but also acceptable wording to avoid stigma, for example by eschewing overly biomedical terminology [13, 14].

Because this adaptation process relies on a set of items identified as important in a different cultural context than the humanitarian setting, you might consider supplementing the tool with additional items that are particularly meaningful for indicating distress or wellbeing locally, such as idioms of distress or resilience [12, 16, 23, 33]. Qualitative research is especially important here.

### Pilot the tool and conduct cognitive interviewing

Before moving on to a formal validation study, it is important to first pilot test adapted tools. Although target community members will have provided feedback on items and recommended particular terms, we have found that shifting from this meta-reflection to having to respond to screening tool items can affect the way that items are understood and responded to [9]. Piloting might point to items that are stigmatized or poorly understood—for example, if almost no one endorses them—or it might indicate where items are not interpreted as intended. We recommend using cognitive interviewing, for example asking participants to “think aloud” as they respond or eliciting what they think the question is asking. This process can reveal additional adaptation that is needed [10].

### Conducting the validation study

This requires that clinicians use a structured clinical interview so that their diagnoses are standardized. In addition, clinicians need to demonstrate that they have inter-rater reliability, which means that they agree when making a diagnosis. Clinicians should be trained on the diagnostic interview; then after practicing, a procedure is needed to establish the inter-rater reliability. If using an alternative approach, such as local idioms of distress, some approach is similarly needed to be sure that people agree on categorizing someone as significantly distressed or not. Otherwise, there is a risk that the idiomatic approach is idiosyncratic to the person nominating others. Moreover, idioms of distress may change over time, differ by educational levels, and have generational variation in interpretation. This can be observed in generational changes over time in English-language tools as well. For example, “feeling blue” was a common idiom in depression tools in the 1960s (e.g., Zhung Self-Rating Depression Scale, Raskin Depression Rating Scale) but is not included in commonly used tools in recent decades (e.g., Patient Health Questionnaire-9). The same idiom may have different interpretations even within the same location, which is why qualitative research to explore idioms of distress is so vital [19, 32].

### Modifying the tool based on the validation results

The validation results not only provide information on overall tool performance but also on individual items. This can be helpful to remove items that do not discriminate between individuals with and without a condition. This may occur for a number of reasons. For example, some symptoms perform poorly in different cultures or because of differences in health status. In Nepal, high rates of gastrointestinal distress led to stomach complaints not being a strong distinguisher between mental health cases and non-cases [15], and this phenomenon has been observed in other settings as well [10]. Doty et al. [3] used item response theory, then analyzed the shorter version of their tools against a structured clinical interview. The shorter versions tended to have slightly lower sensitivity, but specificity increased in most cases. Similarly for the Amharic coherence scale, it may have been helpful to remove items that did not perform comparably to other items, and then evaluate the psychometrics of the shorter version [5].

### Answering other questions for humanitarian mental health

Validation procedures can also be designed to answer a range of other questions relevant to mental health in humanitarian settings. As mentioned above, shortening tools may require item response theory and factor analysis. Factor analysis can also be used to identify whether the conceptual framework for a mental illness has a cultural bias, as Specker and colleagues [27] observed with DSM-5 PTSD domains when working with refugees in Australia. Similarly, large datasets with diverse populations can be used to learn more about cultural equivalence [28]. An especially promising approach is the use of network analysis to pair symptoms with key targets for interventions [21].

## Part 4: Reporting and interpreting validity information for mental health programming

Finally, whether using a previously validated tool or conducting your own validation, it is important to report the validation properties and how they impact interpreting results from program screening or population studies. A key reporting element is to identify the sample on which the tool was validated. At a minimum, it should be in the same language as the target population. However, there are a range of other considerations that can influence comparability, such as the age group, gender distribution, ethnicity or cultural identity, as well as factors such as clinical characteristics (e.g., comorbidities). Similarly, where the validation study was done, e.g., with patients presenting to a clinic vs. a household community survey, will influence the comparability. The next issue to consider is what was the ‘gold standard’ for the validation and how this aligns with what is a meaningful gold standard for the current group. Then, it is vital to report the psychometric properties so others can judge the implications for false positives and false negatives. For example, it is important to report to programmers about the implications of high rates of false positives or false negatives as they relate to achieving program goals. Thus, we recommend reporting all of this information and its implications for interpreting results in a humanitarian context. See Table 2 for a summary of reporting recommendations when considering psychometric properties of an original validation in relation to a new population of interest. For example, a tool validated in Arabic with a community sample of Syrian refugees in Jordan may perform differently when applied to Syrian refugees presenting to primary care clinics in the United States.

**Table 2:** Recommendations for reporting validity information for the original population and implications when applying the tool to a new population of interest

Reporting domain	Validation information	Population of interest	Implications for interpretation
*Population description*	Describe the population with whom the tool was previously validated (e.g., general community, persons with specific traumatic exposures, refugees, torture survivors)	Describe characteristics of the new population being evaluated for humanitarian services	If there are major differences in the original validation group and the new population evaluated in a humanitarian emergency, recommend caution for interpreting new population prevalence rates
*Sampling strategy*	Describe how the validation sample was recruited (e.g., community recruitment, clinical facility recruitment)	Describe how current population will be sampled for evaluation	Convenience and clinical populations likely overestimate prevalence compared to the general population
*Clinical characteristics*	Describe whether the validation sample was help-seeking, had any medical comorbidities (e.g., HIV, disability)	Describe any pertinent clinical aspects of the population of interest	Clinical characteristics may contribute to greater or fewer false positives, e.g., help-seeking populations may have fewer false positives than non-help-seeking groups
*Demographic characteristics*	Describe age, gender distribution, relevant ethnic/ racial/ caste/ or other social groups, economic status	Describe demographics	Cut-off thresholds established with older or younger groups or racial/ethnic majority groups are likely different compared to other age groups or persons from marginalized groups
*Language*	Report language of validation, clarify regional variants, specify literacy level (e.g., illiterate vs. college-educated population)	Report language and literacy levels	Tools validated with higher literacy groups may not be valid when applied to lower literacy groups; validity is impacted when moving between regional variants; if language variant or literacy level changes between original validation and current population, then consider additional qualitative evaluation
*Validation tool*	Describe the gold standard tool used in previous validation	Describe if any validation is done with the new population	Particular gold standard tools may perform better in populations with regular healthcare access for ‘exclusion questions’, e.g., symptoms not attributable to a medical condition
*Prevalence of mental health problem of interest*	Report the prevalence in the validation sample	Report prevalence in the new sample	The greater the difference in population prevalence between the validation sample and new population being evaluated, the greater likelihood of large numbers of false positives or false negatives
*False positive rate*	Report specificity as well as standardized rates (e.g., ratio of false positives to true positives)	Given false positive rate of original validation, report approximate number of false positives in new population	The original validation psychometric properties can be used to provide adjusted prevalence estimates, e.g., providing an adjusted estimate for lower prevalence after accounting for false positive rates
*False negative rate*	Report sensitivity as well as standardized rates (e.g., ratio of false negatives to true positives)	Given false negative rate of original validation, report approximate number of false negatives in new population	The original validation psychometric properties can be used to provide adjusted estimates and number of potential false positives

## Conclusion

In summary, using validated tools in humanitarian settings is vital to know whether persons in need are accurately being identified. Similarly, psychometric properties are particularly informative in low-resource settings where there are financial, time, and human resource costs of including persons in services who do not necessarily need them. Moreover, even when resources are sufficient, including persons in mental health services who do not have a mental health condition may risk stigma or other adverse social consequences. It is important to consider both the purpose of using an assessment tool in a humanitarian setting and conditions of the context when determining what levels of sensitivity, specificity, and other characteristics are needed. Ultimately, the development, adaptation, and validation of assessment tools guided by rigorous qualitative and quantitative methods are needed to ensure that tools function best when serving populations affected by humanitarian emergencies.

## Data Availability

Not applicable.

## Footnotes

1 Although this commentary focuses on validity (are we measuring what we think we are measuring?), psychometric properties related to reliability (are we measuring the outcome consistently between measurements, e.g., between people or over time?) are also important considerations that require their own psychometric determination.
